# Mice with deficiency in *Pcdh15*, a gene associated with bipolar disorders, exhibit significantly elevated diurnal amplitudes of locomotion and body temperature

**DOI:** 10.1038/s41398-024-02952-6

**Published:** 2024-05-28

**Authors:** Daisuke Mori, Chihiro Inami, Ryosuke Ikeda, Masahito Sawahata, Shinji Urata, Sho T. Yamaguchi, Yohei Kobayashi, Kosuke Fujita, Yuko Arioka, Hiroki Okumura, Itaru Kushima, Akiko Kodama, Toshiaki Suzuki, Takashi Hirao, Akira Yoshimi, Akira Sobue, Takahiro Ito, Yukikiro Noda, Hiroyuki Mizoguchi, Taku Nagai, Kozo Kaibuchi, Shigeo Okabe, Koji Nishiguchi, Kazuhiko Kume, Kiyofumi Yamada, Norio Ozaki

**Affiliations:** 1 Department of Psychiatry, Nagoya University Graduate School of Medicine, Nagoya, Japan; 2 Brain and Mind Research Center, Nagoya University, Nagoya, Aichi Japan; 3 Department of Pathophysiology of Mental Disorders, Nagoya University Graduate School of Medicine, Nagoya, Aichi Japan; 4 Department of Neuropsychopharmacology and Hospital Pharmacy, Nagoya University, Graduate School of Medicine, Nagoya, Aichi Japan; 5 Department of Neuropharmacology, Graduate School of Pharmaceutical Sciences, Nagoya City University, Nagoya, Aichi Japan; 6 Department of Otolaryngology, Graduate School of Medicine, The University of Tokyo, Tokyo Pref., Japan; 7 Department of Cellular Neurobiology, Graduate School of Medicine, The University of Tokyo, Tokyo Pref., Japan; 8 Sumitomo Pharma Co., Ltd., Osaka City, Osaka Pref., Japan; 9 Department of Ophthalmology, Nagoya University Graduate School of Medicine, Nagoya, Aichi Japan; 10 Center for Advanced Medicine and Clinical Research, Nagoya University Hospital, Nagoya, Aichi Japan; 11 Medical Genomics Center, Nagoya University Hospital, Nagoya, Aichi Japan; 12 Division of Clinical Sciences and Neuropsychopharmacology, Meijo University Faculty of Pharmacy, Nagoya, Aichi Japan; 13 Division of Behavioral Neuropharmacology, International Center for Brain Science (ICBS), Fujita Health University, Toyoake, Aichi Japan; 14 Division of Cell Biology, International Center for Brain Science, Fujita Health University, Toyoake, Aichi Japan; 15 Institute for Glyco-core Research (iGCORE), Nagoya University, Nagoya, Aichi Japan

**Keywords:** Molecular neuroscience, Bipolar disorder

## Abstract

Genetic factors significantly affect the pathogenesis of psychiatric disorders. However, the specific pathogenic mechanisms underlying these effects are not fully understood. Recent extensive genomic studies have implicated the protocadherin-related 15 (*PCDH15*) gene in the onset of psychiatric disorders, such as bipolar disorder (BD). To further investigate the pathogenesis of these psychiatric disorders, we developed a mouse model lacking *Pcdh15*. Notably, although *PCDH15* is primarily identified as the causative gene of Usher syndrome, which presents with visual and auditory impairments, our mice with *Pcdh15* homozygous deletion (*Pcdh15*-null) did not exhibit observable structural abnormalities in either the retina or the inner ear. The *Pcdh15-*null mice showed very high levels of spontaneous motor activity which was too disturbed to perform standard behavioral testing. However, the *Pcdh15* heterozygous deletion mice (*Pcdh15*-het) exhibited enhanced spontaneous locomotor activity, reduced prepulse inhibition, and diminished cliff avoidance behavior. These observations agreed with the symptoms observed in patients with various psychiatric disorders and several mouse models of psychiatric diseases. Specifically, the hyperactivity may mirror the manic episodes in BD. To obtain a more physiological, long-term quantification of the hyperactive phenotype, we implanted nano tag® sensor chips in the animals, to enable the continuous monitoring of both activity and body temperature. During the light-off period, *Pcdh15*-null exhibited elevated activity and body temperature compared with wild-type (WT) mice. However, we observed a decreased body temperature during the light-on period. Comprehensive brain activity was visualized using c-Fos mapping, which was assessed during the activity and temperature peak and trough. There was a stark contrast between the distribution of c-Fos expression in *Pcdh15*-null and WT brains during both the light-on and light-off periods. These results provide valuable insights into the neural basis of the behavioral and thermal characteristics of *Pcdh15*-deletion mice. Therefore, *Pcdh15*-deletion mice can be a novel model for BD with mania and other psychiatric disorders, with a strong genetic component that satisfies both construct and surface validity.

## Introduction

Extensive epidemiological research, including twin-, adoption-, family-, and population-based studies of increasing quality, has consistently demonstrated significant heritability in all major psychiatric disorders [1]. This evidence suggests that genetic factors are pivotal in predisposing individuals to these conditions. Consequently, genomic studies have been constantly emphasized to elucidate the intricate pathogenic mechanisms associated with these disorders.

Recent genomic investigations have highlighted the protocadherin related 15 (*PCDH15*) gene as a potential key player in the etiology of psychiatric ailments. The relationship between *PCDH15* and the disorders is striking for several reasons. Georgieva et al. genotyped 368 bipolar disorder (BD) and 76 schizophrenia (SCZ) probands, along with their parents. They identified and validated 21 de novo copy number variants (CNVs), including a *PCDH15* deletion in one patient with BD [2]. Another CNV analysis also confirmed *PCDH15* deletions in patients with BD [3]. Our cross-disorder analysis of genic and regulatory CNVs in BD, SCZ, and autism spectrum disorder (ASD) identified *PCDH15* variants in five, one, and two patients, respectively, establishing a link between *PCDH15* deletion and BD pathogenesis [4]. Additionally, cadherin23 (CDH23), which interacts with PCDH15 in their extracellular domains [5], has been linked to SCZ and attention deficit hyperactivity disorder [6–8]. Nevertheless, the precise implications of this protein–protein interaction in neural contexts remain elusive, and the molecular pathogenesis of *PCDH15* in neurological states is yet to be thoroughly explored. Notably, when induced into glutamatergic and GABAergic neurons, induced pluripotent stem cells from patients with BD and a *PCDH15* deletion exhibited both dendritic shortening and a decline in synapse numbers [9].

As a cadherin superfamily member, *PCDH15* contributes to neural differentiation and synapse formation [6]. Both the Human Protein Atlas database and the Allen Mouse Brain Atlas indicate widespread PCDH15 expression in brain regions, such as the cortex, midbrain, cerebellum, and hippocampus, across humans and mice. PCDH15 is highly expressed in human oligodendrocyte progenitor cells. Moreover, it is crucial for their proliferation and significantly influences white matter development and myelination [10]. Consequently, PCDH15 is indispensable in brain neurons and glial cells.

*PCDH15* forms tip-link filaments in sensory hair cells. It is also associated with Usher syndrome type 1 (USH1) [11], the most severe Usher syndrome subtype because of its symptoms such as profound hearing loss, lack of vestibular response, and early-onset retinitis pigmentosa [12, 13]. Notably, there are several reported cases of Usher syndrome in patients presenting with coexisting psychiatric disorders such as BD [14, 15]. However, most Usher syndrome research primarily focuses on retinal and inner ear pathologies, overlooking potential brain pathologies. Unsurprisingly, there is limited knowledge of the relationship between the pathogenesis of BD and other psychiatric disorders and their relationship with *PCDH15*.

Animal models, such as the *Pcdh15*-deletion mice, are crucial for understanding the genetic factors responsible for the development of psychiatric disorders. In the evaluation of animal models for psychiatric disorders, researchers typically consider the following three criteria: (1) surface validity, which reflects the similarities in behavioral features between the animal model and the corresponding human disorder; (2) predictive validity, which assesses the degree to which effective treatments in humans produce similar effects in the model; and (3) construct validity, which considers whether a shared mechanistic theory can explain the animal model and its corresponding human disorder [16–18]. However, despite the abundant animal models developed for psychiatric disorders, few have successfully met multiple validity criteria [19–22].

In this study, we evaluated the behavioral phenotype of *Pcdh15*-deletion mice to establish face validity, ensuring the appropriateness of the model for investigating the mechanisms linking *Pcdh15* deletion to psychiatric disorders such as BD. Additionally, we examined the neural underpinnings of these behavioral traits using whole-brain c-Fos mapping.

## Materials and Methods

### Animal experiments

All research and animal care procedures were ethically approved by the Nagoya University Animal Care and Use Committee. Mice were housed in groups of up to six animals per cage and were maintained under a standard 12-h light/dark cycle (light period: 9:00–21:00) at a constant temperature of 23 °C. Food and water were provided ad libitum.

### Generation of *Pcdh15*-deletion mice

*Pcdh15*-deletion mice were generated using the CRISPR/Cas9 method as previously described [23–26]. Guide RNAs were designed to target exon 5 (NM_001142746.1) of the mouse *Pcdh15* gene. For detailed information, refer to the supplementary material.

### Histological analysis of the inner ear and retina of *Pcdh15*-deletion mice

The inner ear of newborn *Pcdh15-*null was isolated, and cochlear morphology was assessed by staining with a myosin (Myo7A) antibody [27]. Rhodamine phalloidin was used for labeling F-actin. The retinas from 8-week-old *Pcdh15-*deletion mice were isolated, and retinal morphology was evaluated via staining with peanut agglutinin (PNA) or Reversin antibody [28].

### General behavioral analysis

General behavioral analysis of *Pcdh15*-het followed previously established protocols [24, 25] and commenced with the open field test at 8 weeks of age. A series of tests were conducted on 15 male WT and 15 male *Pcdh15***-**het. Data are presented as mean ± standard error. Statistical comparisons between two groups were performed using a two-tailed Student’s t-test. Differences in cliff avoidance reactions were assessed using the Chi-square test [29]. For locomotor activity, PPI test, fear conditioning test, and rota-rod test, repeated analysis of variance (ANOVA) was applied. Multiple group comparisons were analyzed using one-way ANOVA, followed by Tukey’s test when F ratios reached significance (*p* < 0.05). Further details can be found in the Supplementary Materials.

### Measurement of activity and body temperature using a small sensor chip: nano tag

The nano tag (Kissei Comtec Co. Ltd., Nagano, Japan), a device measuring spontaneous locomotive activities and body temperature, was surgically implanted into the abdominal cavity of mice. After surgery, mice were allowed ten days to recover and acclimate to the light–dark cycle. Groups of five mice were housed together in a single cage, with bedding changes and provision of food and water once a week during the light phase. Locomotor activity and body temperature were recorded every 5 min over a continuous 6-week period. Analysis of locomotor activity and body temperature was performed using the nano tag Viewer software (Kissei Comtec Co., Ltd.).

### Mapping of mouse brain activity through automated volume analysis of c-Fos

The analyses aimed to accurately detect, locate, and quantify neuronal activity across the entire brain of the model mice. Each set consisted of three male *Pcdh15*-null at 15 weeks of age and three male WT. Following at least 1 week of synchronization with the light–dark cycle, perfusion fixation was performed at ZT7 or ZT13 using 4% paraformaldehyde/PBS (-). The samples were sent to Certerra Co. Ltd. (USA) for c-Fos expression profiling via immunostaining and light-sheet fluorescence imaging. Automated mapping was conducted using the open-source software ClearMap [30].

For additional materials and methods, please refer to the Supplementary Materials section.

## Results

### Generation of *Pcdh15-*deletion mice

**Fig. 1:**
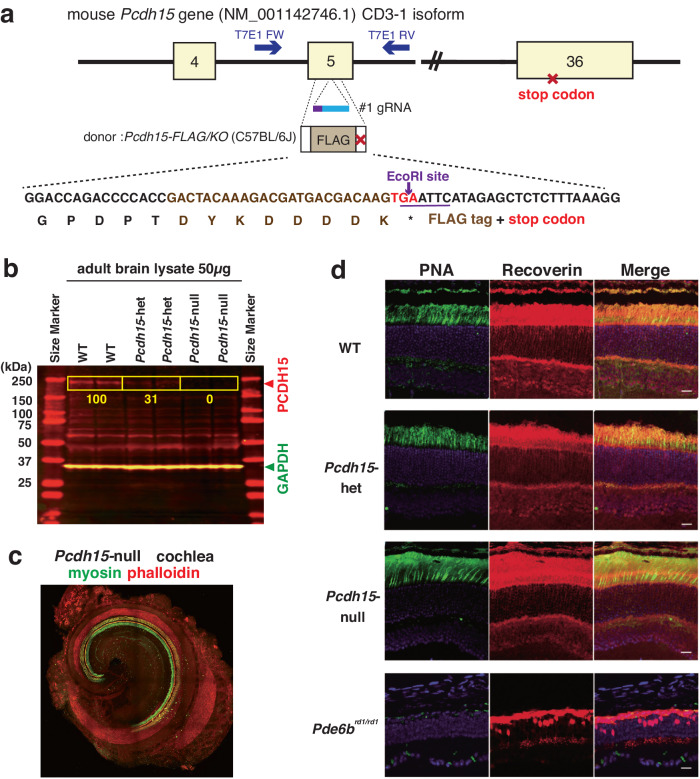
Generation of *Pcdh15*-deletion model mice by the CRISPR–Cas9 system. **a** Schematic representation illustrating the strategy employed for deleting the mouse *Pcdh15* gene using the CRISPR–Cas9 system. The CRISPR–Cas9 knock-in approach aimed to insert a FLAG tag and a stop codon into the *Mus musculus Pcdh15* (NCBI: NM_001142746.1, 9150 bp mRNA) exon 5 (594-754). A specific EcoRI digestion site was introduced solely in the mutant allele to enable genotyping through polymerase chain reaction. **b** Immunoblotting analysis confirming the deletion of full-length PCDH15 in the brains of *Pcdh15-*deletion mice. Each lane contains 50 μg of whole-brain extracts from adult WT, *Pcdh15-*het, and *Pcdh15-*null. Signal detection and quantification were performed using the Odyssey system by LI-COR Biosciences (Lincoln, Nebraska, US). The calculated molecular weight of the PCDH15 CD3 isoform is approximately 183 kDa, denoted by the red signal. The green signal represents the internal control GAPDH. **c** Confocal microscope image showing the structure of the inner ear in *Pcdh15-*null immediately after birth. Myosin (green) and phalloidin (red) co-staining reveals the cochlear morphology. **d** Immunohistochemical staining of the retina from *Pcdh15-*null. Retinas from 4-week-old *Pcdh15-*null, *Pcdh15-*het, and WT were stained with peanut agglutinin (PNA; green) and Recoverin (red). Normal formation of the photoreceptor and bipolar cell layers is observed in all genotypes, compared with Pde6b^rd1/rd1^ mice that display retinal degeneration [28]. *Pcdh15* protocadherin related 15, *Pcdh15*-het *Pcdh15* heterozygous deletion mice, *Pcdh15*-null *Pcdh15* homozygous deletion mice, WT wild-type mice.

To elucidate the phenotypic abnormalities associated with the onset of mental disorders observed in *PCDH15*-deletion subjects, we employed CRISPR–Cas9 (clustered regularly interspaced short palindromic repeats and CRISPR-associated protein 9) genome editing technique to generate a genetically modified mouse model (Fig. 1a) [26]. The conservation of the *Pcdh15* gene structure between humans and mice has been well established [31]. Leveraging this fact, we designed CRISPR-targeted guide RNAs and single-stranded DNA to facilitate the insertion of a FLAG-tag sequence and a stop codon into exon 5, thus creating the *Pcdh15-*deletion model mouse (Fig. 1a and S1a). The #1 guide RNA was chemically synthesized as a CRISPR RNA, and trans-activating CRISPR RNA (tracr-RNA), Cas9 protein, and donor DNA were co-microinjected into 121 fertilized eggs in C57BL/6J mice breeding in Charles River Inc (Wilmington, Massachusetts, US) and cultured until the two-cell stage. Of these eggs, 75 were subsequently transplanted into the oviducts of pseudopregnant mice, resulting in the birth of 19 littermates. Ultimately, through polymerase chain reaction cloning, the successful generation of two knock-in mice was confirmed according to the design (Fig. S1b). For conventional genotype screening of the model mice, primers were meticulously designed to distinguish between wild-type mice (WT) and mutant (Flag-KO) alleles via agarose gel electrophoresis (Fig. S1c).

Immunoblot analysis was performed to validate the protein expression level of PCDH15 in the newly generated *Pcdh15*-deletion mice (Fig. 1b). In the adult brain, PCDH15 expression in *Pcdh15*-het was 31% of that measured in WT, whereas PCDH15 was scarcely detectable in *Pcdh15*-null. Employing the PCDH15-specific antibody, immunoblotting on major organs of WT revealed discernible signals solely in the brain (Fig. S1d). Furthermore, the presence of PCDH15 protein was evident in the brain throughout developmental stages into adulthood, with a clear peak during the initial 3 days after birth (Fig. S1e). In the *Pcdh15-*null allele, because of the study design, FLAG tags were added in frame. Attempts were made to confirm the presence or absence of a truncated FLAG protein using immunoblotting; however, it could not be detected with the widely used anti-FLAG antibody (M2 mouse monoclonal antibody, Sigma-Aldrich, US) (data not shown). Within primary cultured neurons, the expression of PCDH15 protein increased as the neurons matured (Fig. S1f). *Pcdh15-*null exhibited normal birth and growth patterns. Detailed examination of their brains revealed the absence of significant structural anomalies (Fig. S1g). Considering that retinal and inner ear structural abnormalities have been reported in spontaneously occurring *Pcdh15*-deletion mice [32], we meticulously examined the pathology of our *Pcdh15*-null and found normal structural features (Figs. 1c and d).

### General behavioral analysis of *Pcdh15*-het

**Fig. 2:**
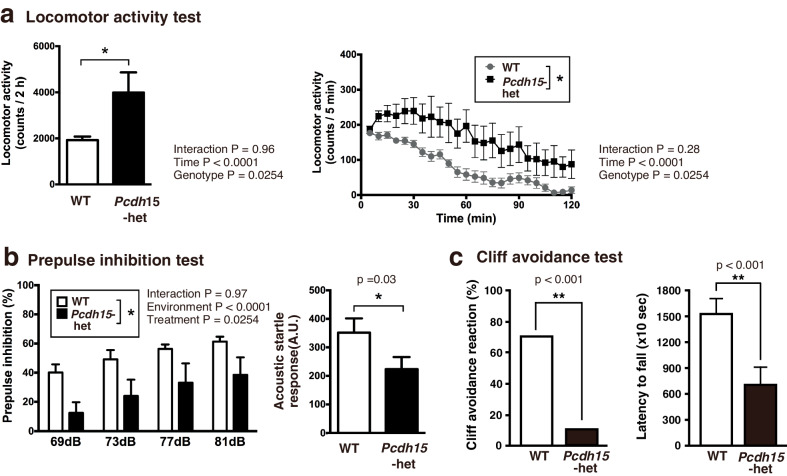
Behavioral analyses of *Pcdh15*-het. **a** Locomotor activity test results depicting total activity during a 2-h measurement period (left), and activity recorded every 5 min (right). **b** Results of the prepulse inhibition (PPI) test. *n* = 15 for male WT mice, *n* = 15 for male *Pcdh15*-het in panels a and b. The data of locomotor activity test and PPI were analyzed using a two-way ANOVA. **c** Cliff avoidance test outcomes illustrating the percentage of cliff avoidance reaction (left) and the latency of falling from a cliff (right). *n* = 14 for male WT, *n* = 14 for male *Pcdh15*-het in panel 2c. Data are presented as the mean ± standard error of the mean (SEM). Student’s *t*-test was used for these analyses. ∗∗*p* < 0.01; ∗*p* < 0.05 indicate statistical significance. For additional information, please refer to Table 1.

*Pcdh15*-null displayed marked hyperactivity (Movie S1). Consequently, comprehensive behavioral assessments were exclusively conducted on *Pcdh15*-het, which exhibited elevated locomotor activity and reduced prepulse inhibition (PPI) (Figs. 2a, b, and S2; Table 1). Additionally, in the cliff avoidance test, a metric for assessing impulsivity, *Pcdh15*-het spent significantly shorter time to jump off the platform and exhibited an absence of cliff avoidance response (Fig. 2c). There were no significant changes observed between WT and *Pcdh15*-het (Fig S2 and Table 1) in the open field, Y-maze, elevated cross maze, rotarod, novel object recognition test, social interaction, and fear conditioning test.

**Table 1:** Comparison of behavioral changes between WT mice and the Pcdh15 heterozygous deletion mutant in each test.

Test	parameter	Result
Open-field test	Inner zone (time) :	ND
	Outer zone (time) :	ND
Elevated plus maze test	Open arm (time) :	ND
	Closed arm (time) :	ND
	Open arm (counts) :	ND
	Closed arm (counts) :	ND
	Total (counts) :	ND
Light dark transition test	Light box (time) :	ND
	Dark box (time) :	ND
Y-maze test	Alternation behavior (%) :	ND
	Total arm entry:	ND
Novel object recognition test	Exploratory preference (%) :	ND
	Total exploratory time :	ND
Fear conditioning test	Training (time) :	ND
	Contextual (time) :	ND
	Cue (time) :	ND
Rota-rod test	Training (time)	ND
	Test (time)	ND
Locomotor activity test	Locomotor activity (time course) :	↑
Social interaction test	Habituation (empty) :	ND
	1st session (stranger 1) :	ND
	2nd session (stranger 1 + 2) :	ND
Prepulse inhibition test	PPI (%) :	↓
	Startle response :	↓
Cliff avoidance test	Cliff avoidance reaction (%) :	↓
	Latency to fall (x10 sec) :	↓

Behavioral tests were performed using 15 male WT mice and 15 male Pcdh15-het mice. All data are reported as the mean ± standard error of the mean (SEM) and were analyzed using the Prism software. The data from three-chambered social-interaction, PPI, locomotor, rota-rod, elevated plus maze, novel object recognition, and fear-conditioning tests were analyzed using the two-way ANOVA with or without repeated measures, followed the post-hoc Bonferroni test. The data obtained for PPI (Acoustic startle response), locomotor (Locomotor activity), rota-rod (Test phase), open field, Y-maze, and fear-conditioning (contextual) parameters were assessed using the Mann–Whitney *U* test.

↑: higher than the WT, ↓: lower than the WT, ND: no difference

### Elevated locomotion and body temperature amplitudes in *Pcdh15*-null

We focused on the hyperactivity of *Pcdh15*-deficient mice. In a 24-h locomotor activity test conducted on *Pcdh15*-WT, *Pcdh15*-het, and *Pcdh15*-null mice, the *Pcdh15*-null mice exhibited intense activity during the dark phase (Fig. S3). Moreover, to observe the characteristics of *Pcdh15*-deletion mice under more physiologically relevant conditions, we conducted continuous measurements of spontaneous locomotion and body temperature in both *Pcdh15*-null and WT. Thus, we introduced a small accelerometer chip (nano tag^®^) into the abdominal cavity of each mouse. One week after implantation, the mice were allowed to acclimate to an environment with alternating light-on/off periods set at Zeitgeber time 0 (ZT0) (9:00) and ZT12 (21:00) for an additional week. Thereafter, the measurement phase commenced.

**Fig. 3:**
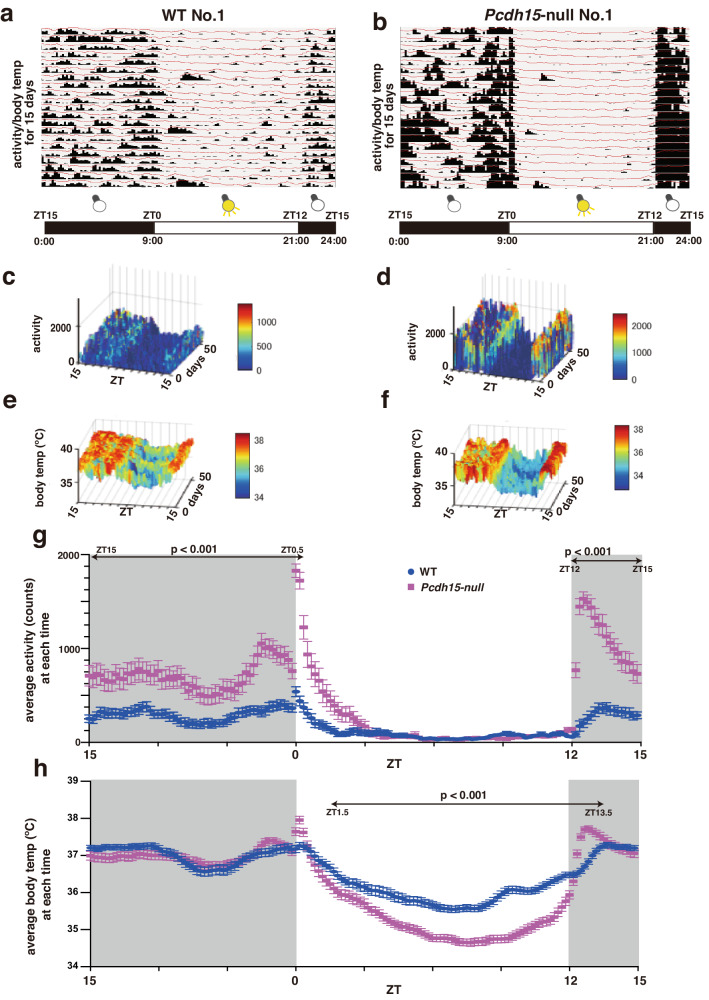
Long-term activity and body temperature measurement with a miniature accelerometer: nano tag. **a**, **b** The presented data spans the light–dark (L–D) cycle of a mouse model, where the nano tag was intraperitoneally implanted and visualized through the nano tag Viewer software. Data for the first 15 days are displayed. The horizontal axis represents the time from ZT15 (00:00) to ZT15 (00:00) of the following day. The vertical axis histograms indicate activity levels per 5 min, whereas the line graphs depict body temperature. **a** represents WT No. 1 and **b** represents *Pcdh15-*null No. 1. Data for the other WT and *Pcdh15*-null (No. 2, 3, and 4) are shown in Supplementary Figure 4. **c**–**f** Three-dimensional surface plots created using MATLAB software (The MathWorks Inc., Natick, Massachusetts, USA) illustrate the activity and temperature fluctuations across time for the entire monitoring period. The color bars denote the intensity of each parameter: **c** activity in WT; **d** activity in *Pcdh15*-null; **e** body temperature in WT; and **f** body temperature in *Pcdh15*-null. **g**, **h** Mean ± SEM of 6-week data obtained from four WT and four *Pcdh15*-null; the data were measured throughout the L–D cycle and plotted against time. The vertical axis represents activity (**g**) or body temperature (**h**) in 10-min intervals, whereas the horizontal axis depicts time within a 24-h period. A statistical analysis of the activity levels or body temperature at each time point was conducted via multiple *t*-tests using the GraphPad Prism 8 software, indicating significant time intervals with *P* < 0.001. In terms of activity levels, *Pcdh15*-null mice exhibited increased activity compared with the controls at all time points from lights off until 30 min after the lights were turned back on. Regarding body temperature, significant differences were observed at all time points from 1 h 30 min after lights on to 1 h 30 min after lights off. ZT Zeitgeber time.

The activity profiles distinctly revealed the nocturnal nature of both mouse groups, with *Pcdh15*-null exhibiting substantially higher levels of activity (Figs. 3a, b, S4a, and b). Mesh plots were generated for time, activity and body temperature, and days over the course of 6 weeks of continuous monitoring (Figs. 3c–f). The pronounced surge in activity during the light-off period in *Pcdh15*-null remained statistically significant across the entire duration of measurement (Figs. 3c, d, g, S4c, and d). These data also include activity noise generated by weekly changes in bedding. In terms of body temperature, the difference between the highest and lowest body temperature within a single day for *Pcdh15*-null was 1.77 times greater than that of WT (WT: 1.736 °C; *Pcdh15*-null: 3.072 °C; Fig. 3h, S4e, and f).

Furthermore, analysis of the temporal patterns showed that both WT and *Pcdh15-*null exhibited their lowest body temperature around ZT7 (16:00) and their highest body temperature around ZT13 (22:00). These patterns were consistently observed throughout the entire monitoring period (Figs. 3e, f, h, S4e, and f). *Pcdh15*-null did not show significant circadian anomalies (Fig. S5a). Their daily rest duration remained consistent with that of the WT, despite their elevated activity levels (Figs. S5b and c).

### c-Fos mapping in *Pcdh15*-null whole brain

To elucidate the underlying neural circuitry responsible for the behavioral phenotypes and distinctive diurnal temperature fluctuations in *Pcdh15*-deletion mice, we thoroughly investigated the variations in expression levels of neural activity markers across the entire brain of both WT and *Pcdh15-*null using c-Fos mapping at two specific time points, namely light-off and light-on [33, 34].

**Fig. 4:**
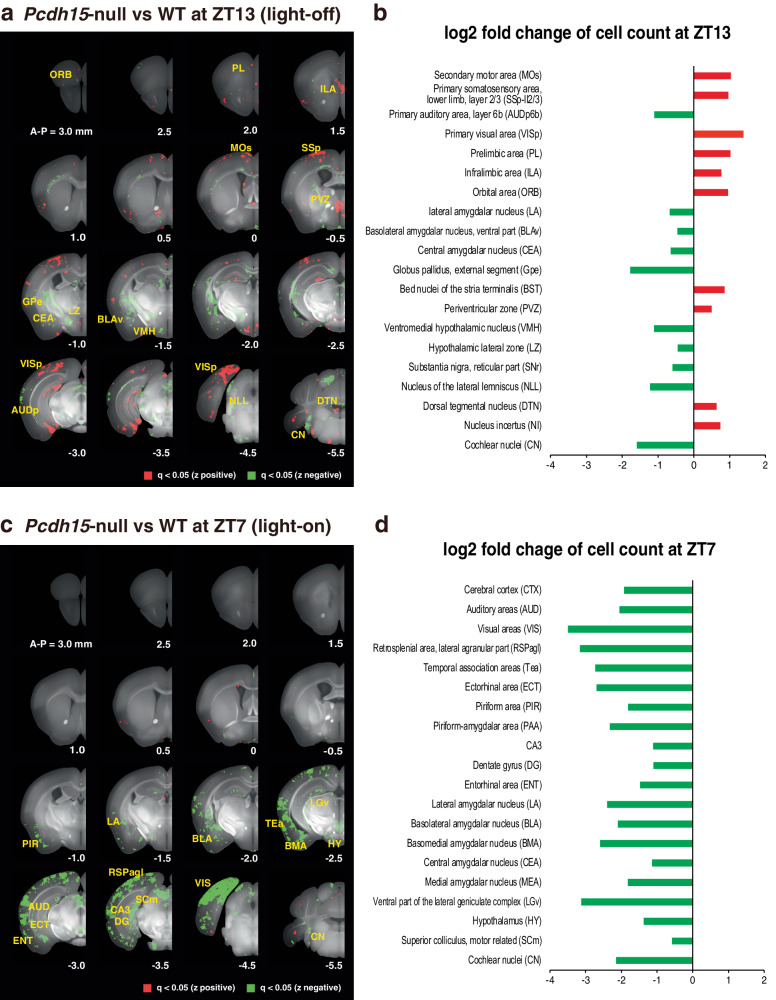
c-Fos mapping in the *Pcdh15*-null whole brain. **a**–**d** Results of automated analysis of c-Fos-positive cell distribution in mouse brains collected at ZT13 or ZT7. *n* = 3 for WT and *n* = 3 for *Pcdh15-*null. q value voxel maps comparing WT with *Pcdh15*-null (**a**) at ZT13 and (**c**) at ZT7. A-P: distance from bregma in mm. Bar plots illustrating the log2 fold-change in each brain region (**b**) at ZT13 and (**d**) at ZT7. Red voxels and bars indicate *q* < 0.05 and z > 0. Green voxels and bars indicate *q* < 0.05 and z < 0.

At ZT7 or ZT13, we conducted perfusion and fixation procedures for both WT and *Pcdh15*-null, followed by brain removal and transparency enhancement to facilitate whole-brain c-Fos mapping [35]. During ZT13, *Pcdh15*-null exhibited areas of elevated and diminished c-Fos expression compared with those of WT (Fig. 4a and b). Notably, the regions displaying elevated c-Fos expression during ZT13 in *Pcdh15*-null were primarily situated in the cortex, encompassing specific areas such as the primary somatosensory region, secondary motor regions, and primary visual region (Fig. S6a). Conversely, at ZT7, there was a significant reduction in c-Fos expression across the entire cortex in *Pcdh15-*null, with no regions indicating increased c-Fos expression (Figs. 4c and d). Interestingly, within the amygdala, including the central amygdalar nucleus and basolateral amygdalar nucleus, c-Fos expression was consistently impaired during both ZT13 and ZT7. Moreover, decreased c-Fos expression was observed in the hypothalamus, an important center for thermoregulation, of *Pcdh15*-null during ZT7.

## Discussion

In this study, we demonstrated that *Pcdh15*-deletion mice display traits consistent with construct and surface validities, rendering them reliable models for the investigation of psychiatric disorders such as BD. The conclusions are based on observable phenotypic characteristics, such as hyperactivity, decreased PPI, and increased impulsivity, in these mice. The regulation of body temperature is a core biological function affected by circadian rhythms. However, this is known to be disturbed in BD [36] and the expected dysregulation was observed. Our extensive nano tag measurements over a prolonged period revealed a significant diurnal variation in body temperature in the *Pcdh15*-deletion mice. We further elucidated the neural mechanisms underlying these phenotypic traits by performing comprehensive c-Fos mapping across the entire brain.

### *Pcdh15*-deletion model mice compared with *av* mice: model of Usher syndrome

Via a whole-genome analysis targeting Japanese patients with psychiatric disorders, such as BD, we identified multiple patients with a loss of the protein-coating-encoding region of the *PCDH15* gene [4]. According to various databases, such as UniProt, the *PCDH15* gene exhibits diverse isoforms. Three types of intracellular domains, CD1, CD2, and CD3, are well known on the C-terminal end of the protein product. Furthermore, the cell-guidance domain located on the N-terminal side is highly diverse. Therefore, rather than mimicking specific patients, we aimed to focus on exons encoding a greater number of proteins reported in the database, and aimed to nullify *PCDH15*. Particularly, the CD3 isoform (UniProt: Q99PJ1-18) is abundantly expressed in the brain. Exon 5 of Q99PJ1-18 has a length of 161 bp, which is not a multiple of three, and it was selected because a suitable guide RNA for CRISPR/Cas9 cleavage could be designed.

Usher syndrome type 1 (USH1) is a genetic disorder that is characterized by severe hearing loss and retinitis pigmentosa [36]; furthermore, variants in the *Pcdh15* gene have been identified in families with Usher syndrome type 1F (USH1F) [11]. The mouse Ames waltzer hearing-loss mutant (*av*) exhibits a phenotype associated with hearing loss and balance disorders caused by degeneration of the inner ear neuroepithelium, with the *Pcdh15* gene being identified as the causative gene of this condition [37]. RT-PCR results showed that the av mutant mice *av-J*, *av-2J*, and *av-3J* are all incapable of expressing the full-length PCDH15 protein [37]. Similarly, our novel *Pcdh15-*deletion mice did not express the full-length PCDH15 protein, as confirmed via immunoblot analysis (Fig.1d), thus sharing a commonality with the *av* mice.

*PCDH15* gene expression is restricted to the brain and inner ear [37]. Our *Pcdh15-*null mice did not exhibit lethality. *Pcdh15*^av-2J^ mice carry a spontaneous mutation at the *Pcdh15* locus and are characterized by head tossing, circling, and deafness [37]. Severe head tossing and circling were also observed in our *Pcdh15-*null mice (Fig. S2, Movie S1). PCDH15 expression has been identified by immunostaining the stereocilia of cochlear hair cells and retinal photoreceptors, which are presumed to be necessary for their formation and maintenance [32]. It was anticipated that the loss of PCDH15 would result in fatal structural abnormalities in the inner ears and retinas of *Pcdh15-*deficient mice. However, no such macroscopic structural abnormalities were observed in either the inner ears or retinas of the *Pcdh15-*deficient mouse model (Fig. 1c and d). Thus, there may be a need to investigate more-subtle structural and functional abnormalities.

The cause of the phenotypic differences observed between *Pcdh15-*deficient mice and the *av* lines is unknown; however, they may be attributed to variations in the cleavage of the PCDH15 protein/mRNA product. Using immunoblotting, we confirmed the complete loss of the full-length PCDH15 protein (Fig. 1b, S1d). The PCDH15 signal is also lost in the immunostaining of *av* mice [38]. Higher organisms, including mice, exhibit a wide array of alternative splicing isoforms in both the extracellular and intracellular regions of the *Pcdh15* gene; hence, disparities in the impact of incomplete gene products caused by disrupted exons may arise.

### Validity of *Pcdh15*-deletion mice as a psychiatric model

Historically, most studies on animal models of psychiatric disorders began by identifying animal behaviors mirroring human pathology, focusing primarily on surface validity. Subsequent studies explored predictive and construct validities [39]. However, models involving rare CNVs that are generally associated with a high risk of psychiatric disorders have recently emerged [40, 41]. Notably, these models have distinctly prioritized the establishment of construct validity from the outset. Similarly, we attempted to create a mouse model of psychiatric disease that meets the criteria for construct validity. This was accomplished through the targeted deletion of *Pcdh15*, a gene recognized in patients with psychiatric disorders. Subsequently, we evaluated the surface validity of this model. Our findings indicate that *Pcdh15*-deletion mice display phenotypes consistent with those observed in analogous disease models [42–45] and patients [46–50]. Consequently, *Pcdh15*-deletion mice appear to be a promising model for psychiatric disorders, satisfying both construct and face validity criteria.

We have previously highlighted the significant association of *Pcdh15* deletion with BD [4]. Certain phenotypes, such as the hyperactivity noted in *Pcdh15*-deletion mice, may mirror the manic symptoms of BD. Whole-brain c-Fos mapping has shown altered neural activity in the amygdala, a region where structural abnormalities frequently occur in adult patients with BD or SCZ [51–53]. The consistent dysfunction of the amygdala implies that *Pcdh15*-deletion mice may also satisfy surface validity as a model of psychiatric disorders in terms of brain function.

Throughout 6 weeks of extended observations using nano tags, *Pcdh15*-null consistently displayed manic (hyperlocomotive) behavior rather than depressive (hypolocomotive) tendencies. A comparison can be drawn with the mitochondrial DNA polymerase catalytic subunit (*Polg1*) mutant mice, a model for BD, where multiple episodes of inactivity were detected over a 6-month observation period [54]. To enhance the face validity of *Pcdh15*-deletion mice in modeling BD, extending the observational period using nano tags might help capture both manic and depressive episodes, thereby characterizing the cyclical nature of BD.

### Phenotypes of *Pcdh15*-deletion mice

BD, which is genetically linked to *PCDH15* deletion, manifests as recurring depressive and manic episodes [55]. Manic episodes are marked by symptoms such as euphoria, aggression, sleep disruptions, elevated reward-seeking behavior, hypersexuality, and hyperactivity [56, 57]. Our tests revealed *Pcdh15*-het displayed hyperactivity (Fig. 2a), diminished PPI (Fig. 2b), and increased impulsivity (Fig. 2c). These findings mirror those of previously documented mouse models of mania [42, 44] and patients with BD [48–50]. Some overlapping phenotypes have been observed in patients with ASD and SCZ or animal models with *Pcdh15* deletion [43, 45–47].

However, behavioral tests can be skewed by various external factors, such as the testing environment or researcher [58, 59]. The behavior of mice outside the home cage or when isolated can be erratic. The importance of conducting long-term behavioral observations in a less constrained and more natural environment has also been recognized [60]. In our analysis, we employed the nano tag system to address these issues [61, 62]. Despite its significant weight in relation to the mice, the nano tag successfully highlighted the hyperactive tendencies in *Pcdh15*-null across 6 weeks in their home cages. We demonstrated similar results on a 24-h continuous locomotor activity test by assessing activity based on the number of crossings of an infrared beam (Fig. S3). The hyperlocomotive behavior of *Pcdh15*-null was more pronounced immediately after light-off and subdued after light-on (Fig. 3 and S4). Patients with psychiatric disorders, including BD, often suffer from sleep disturbances, including circadian rhythm disruptions [63]. However, *Pcdh15-*null mice did not exhibit significant circadian rhythm abnormalities (Fig. S5a). Despite the increased activity levels, their daily rest hours were not significantly different from those of WT (Figs. S5b and S5c). While activity-based sleep–wake rhythm analysis is common in rotating cages [64], comprehensive studies integrating electroencephalography and other methodologies are required to fully understand the effect of *Pcdh15* deletion on sleep.

Another key feature of the nano tag is its ability to measure core body temperature. In our study, *Pcdh15*-null exhibited their lowest body temperature around ZT7 during the light-on period. The values peaked at ZT13 after light-off (Fig. 3h). *Pcdh15*-null showed a diurnal temperature amplitude that was 1.7 times greater than that of WT. The hypothalamus acts as the thermoregulatory center of the brain [65]. The periventricular zone, which showed increased c-Fos expression at ZT13 in *Pcdh15*-null, is a hypothalamic region containing the paraventricular hypothalamic nucleus, which is part of a neural circuit essential for emotion and thermoregulation [66]. The temperature increase toward ZT13 may be attributed to the activation of the paraventricular hypothalamic nucleus. Conversely, at ZT7, *Pcdh15*-null exhibited reduced c-Fos expression in the hypothalamus, which includes the dorsomedial nucleus of the hypothalamus (q = 5.37 × 10^−9^), a vital component of the previously mentioned neural circuit. The exact mechanism responsible for the fluctuations in temperature due to *Pcdh15* deletion remains elusive. Hence, there is a need for a thorough examination of the interplay between the hypothalamic regions and metabolism.

### Relationships between phenotypes and brain activity in *Pcdh15*-null

c-Fos is a transcription factor robustly induced by neural activity, with expression peaking within 5–10 min of stimulation and lasting 2–3 h [67–69]. Consequently, it serves as a reliable marker of recent neural activity [68, 70].

In this investigation, c-Fos mapping illuminated the brain activity landscape in *Pcdh15*-null. At ZT13, *Pcdh15*-null brains exhibited increased c-Fos expression in the primary visual cortex, prefrontal cortex, and orbital regions compared with those of WT. In contrast, they showed decreased levels in the amygdala, globus pallidus, and certain hypothalamic regions (Fig. 4a and b). However, at ZT7, numerous regions in *Pcdh15*-null brains showed reduced c-Fos expression (Figs. 4c and d). Notably, *Pcdh15*-null brains at both ZT7 and ZT13 were characterized by decreased c-Fos levels in the amygdala, a pivotal region for emotions such as fear and anxiety [71]. This reduced activity could mirror emotional and interpersonal deficits observed in psychiatric disorders [72]. Based on magnetic resonance imaging findings suggesting a structurally reduced amygdala in BD and SCZ [51–53], a more comprehensive exploration of this aspect in *Pcdh15*-null is warranted. The Yakovlev circuit is centered in the amygdala and links the orbitofrontal cortex, insula, and anterior temporal lobe; it underpins emotions and motivation [73, 74]. In *Pcdh15*-null at ZT13, alterations in c-Fos expression were observed in multiple brain regions encompassed by this circuit, extending beyond the amygdala. This suggests that disruptions in this circuitry could potentially contribute to the manifestation of symptoms related to psychiatric diseases.

Correlating c-Fos mapping to observed phenotypes, c-Fos expression was elevated at ZT13 (the active phase) in both WT and *Pcdh15*-null compared with that at ZT7 (Fig. S6b). This surge in the hyperlocomotive *Pcdh15*-null at ZT13 may be ascribed to increased physical activity [75]. This hypothesis aligns with findings indicating that running elevates c-Fos in areas such as the primary somatosensory and secondary motor cortex [76].

*Pcdh15* deletion has also been linked to diminished PPI (Fig. 2b). While the neural circuits governing PPI have been identified [77], it is established that inhibition of the globus pallidus dampens PPI [78]. In our study, the globus pallidus in *Pcdh15*-null at ZT13 showed reduced c-Fos expression. The auditory cortex, another PPI player [79], exhibited decreased c-Fos levels in *Pcdh15*-null at ZT13.

Furthermore, *Pcdh15* deletion correlated with increased impulsivity (Fig. 2c). The orbitofrontal cortex is implicated in impulsivity [80] and structurally altered in patients with BD [81]. It interacts with the amygdala, thereby suppressing its activity [82]. This interplay in *Pcdh15*-null at ZT13 potentially underlies the increased impulsivity and other psychiatric symptoms.

Comparison of WT and mutant mice under consistent conditions revealed variations in c-Fos expression across brain regions. Immunostaining of the primary visual cortex confirmed our c-Fos mapping findings (Fig. S6c). Furthermore, analysis of samples obtained from around this cortex through reverse transcription-polymerase chain reaction revealed congruent c-Fos mRNA levels (Figs. S6d and e). These findings affirm the utility of c-Fos brain mapping for analyzing neural activity in mutant mice and considering the neural circuit basis of the phenotype.

This study had some limitations. First, this model lacks validation regarding the predictive validity of BD. Second, the effects of treatment using existing therapeutic agents on the activity and body temperature need to be investigated. In the future, as more comprehensive genomic analysis data of patients with psychiatric disorders, including BD, becomes available, there is a sufficient possibility that the genetic association between the *PCDH15* variants and onset could become statistically significant from a genetic standpoint.

In conclusion, the results of our study revealed that *Pcdh15*-deletion mice display key phenotypes, including hyperactivity. Our comprehensive analysis of whole-brain activity provided significant insights into the neural circuitry underlying these phenotypes. Considering these findings, *Pcdh15*-deletion mice emerge as a potentially valuable model, meeting both construct and surface validity criteria, for investigating the mechanisms linking *PCDH15* deletion to psychiatric disorders, notably BD with mania.

### Supplementary information

Supplementary Materials

Supplementary Movie 1

## Data Availability

The datasets generated during and/or analyzed during the current study are available from the corresponding author upon reasonable request.
