# Supplementary material for: Mice with deficiency in Pcdh15, a gene associated with bipolar disorders, exhibit significantly elevated diurnal amplitudes of locomotion and body temperature
Source: Transl Psychiatry. 2024 May 28;14:216. doi: 10.1038/s41398-024-02952-6 (PMC11133426; doi:10.1038/s41398-024-02952-6)
Supplement: Supplementary file 1 — Supplementary Materials [file 41398_2024_2952_MOESM1_ESM.docx]

**Mice with deficiency in *Pcdh15*, a gene associated with bipolar disorders, exhibit significantly elevated diurnal amplitudes of locomotion and body temperature**

**Supporting Information**

**Table of Contents**

**I. Supplementary Methods**

**Generation of *Pcdh15-*deletion mice**

**Immunoblots**

**Behavioral analysis**

**24-h locomotor activity test**

**Hematoxylin and eosin (HE) staining**

**Immunohistochemistry**

**Phase analysis**

**Mapping of mouse brain activity through an automated volume analysis of c-Fos**

**Preparation of total RNA**

**RT-PCR**

**Quantitative RT-PCR (qPCR)**

**II. Supplementary Figures**

**Figure S1.** Generation of *Pcdh15*-deletion mice and expression profiles of PCDH15 protein.

**Figure S2** General behavioral analysis of *Pcdh15-*het.

**Figure S3** Twenty-four-hour locomotion activity test.

**Figure S4** Successive data on activity and body temperature in mice implanted with nano tag in the abdominal cavity.

**Figure S5** Analyses of locomotor activity using nano tag.

**Figure S6** Various studies on c-Fos expression in the brain.

**Table S1** Comparison of c-Fos-positive cell counts between WT and *Pcdh15*-null at ZT13

**Table S2** Comparison of c-Fos-positive cell counts between WT and *Pcdh15*-null at ZT7

**Movie S1** *Pcdh15-*null showing marked hyperlocomotion.

**III. Supplementary References**

**I. Supplementary Methods**

**Generation of *Pcdh15-*deletion mice**

**(i) CRISPR/Cas plasmid**

A pair of oligo DNAs (Invitrogen, USA) corresponding to the *Pcdh15* single-guide RNA were hybridized and ligated using T4 DNA ligase (TOYOBO, Japan) into the linearized pSpCas9(BB)-2A-GFP (PX458) plasmid (Addgene, #48138; Feng Zhang, MIT), which was digested with BbsI (NEB), as previously described [1, 2]. The DNA primers utilized in this study are listed below.

| PX458-gRNA construction | | 5' to 3' sequence |
| --- | --- | --- |
| mPcdh15 #1 gRNA | Forward | CACCGTTAAAGAGAGCTCTATGGTG |
|  | Reverse | aaacCACCATAGAGCTCTCTTTAAC |
| mPcdh15 #2 gRNA | Forward | CACCGCTCTCTTTAAAGGACAACG |
|  | Reverse | aaacCGTTGTCCTTTAAAGAGAGC |
| mPcdh15 #3 gRNA | Forward | CACCGGAAAAGCATCTGTTTAACG |
|  | Reverse | aaacCGTTAAACAGATGCTTTTCC |
| mPcdh15 #4 gRNA | Forward | CACCGATGCTTTTCCTGAACAGTAC |
|  | Reverse | aaacGTACTGTTCAGGAAAAGCATC |
| β-actin gRNA (positive control) | Forward | caccGCATTATGAGTCCTTAAGTGA |
|  | Reverse | aaacTCACTTAAGGACTCATAATGC |
|  |  |  |
| T7E1 assay and Genotyping | | 5' to 3' sequence |
| mPcdh15 | Forward | GAGTATCAAATTGATCACCTCAGACTCAG |
|  | Reverse | GCCTCACTGAGTAGATTTGGTATCCTG |
| β-actin | Forward | GACTGGGATCATTTGTTCACACATGCAG |
|  | Reverse | GGCCCTTGAACTTCGGTATCTACACTGAG |

**(ii) T7 Endonuclease I (T7E1) assay**

The T7E1 assay was conducted in mouse Neuro2A cells following previously established protocols. [1, 2] Briefly, Neuro2A cells were transfected with *Pcdh15*-pX458 plasmids or empty pX458 plasmids in a 12-well plate using Lipofectamine 3000 (Life Technologies). Genomic DNAs were isolated 48 hours post-transfection by digestion with proteinase K (P2308, Sigma-Aldrich, USA) followed by ethanol precipitation. Subsequently, *Pcdh15* loci were PCR amplified from the purified genomic DNA using the following primers: 5'-GAGTATCAAATTGATCACCTCAGACTCAG-3' and 5'-GCCTCACTGAGTAGATTTGGTATCCTG-3'. The PCR products were subjected to slow annealing and then digested with the T7E1 enzyme at 37°C for 30 min. The resulting products were analyzed through electrophoresis on a 2% agarose gel.

**(iii) Chemical synthesis of crRNA and tracrRNA**

*Pcdh15*-CRISPR RNA (crRNA) (5′- UUAAAGAGAGCUCUAUGGUGguuuuagagcuaugcuguuuug-3′) and trans activating RNA (tracrRNA) (5′- AAACAGCAUAGCAAGUUAAAAUAAGGCUAGUCCGUUAUCAACUUGAAAAAGUGGCACCGAGUCGGUGCU-3′) were chemically synthesized and purified using polyacrylamide gel electrophoresis (Fasmac, Atsugi, Kanagawa, Japan).

**(iv) Injection**

Injection mixtures were prepared following a previously established method. [3] To produce Pcdh15 knockout mice through protein injection, a combination of Cas9 proteins, Pcdh15-crRNA, tracrRNA, and donor single-strand DNA (ssDNA) was prepared in Tris-EDTA buffer, achieving working concentrations of 100 ng/μL, 0.61 pmol/μL, 0.61 pmol/μL, and 10 ng/μL, respectively. Cas9 proteins were sourced from NEB (M0386S, USA). The donor ssDNA, encoding the FLAG-tag and stop codon and targeting exon 5 of the mouse Pcdh15 gene (NM_001142746.1), was chemically synthesized by Fasmac Inc. (Japan). The mixture was then incubated at 37°C for a minimum of 15 min before being injected into the pronuclei of one-cell-stage zygotes obtained from the C57BL/6J strain (Charles River, USA).

**(v) PCR screening and genotyping**

For the purpose of PCR screening of Pcdh15-het embryos resulting from the injection of CRISPR mix into zygotes, genomic DNAs were extracted from tails using proteinase K digestion, followed by a standard phenol extraction method. Pcdh15-het embryos were screened using PCR with KOD FX Neo (TOYOBO), and the resulting products were analyzed by electrophoresis in a 2% agarose gel. Primer sequences for gene detection were as follows: 5'-GAGTATCAAATTGATCACCTCAGACTCAG-3' and 5'-GCCTCACTGAGTAGATTTGGTATCCTG-3'. Furthermore, PCR products were further cloned using the Zero Blunt TOPO PCR Cloning Kits (Life Technologies) and subjected to sequencing analysis, as described previously. [1, 2] After the establishment of mouse strains, conventional screening can be carried out through PCR, followed by EcoRI restriction enzyme digestion of the PCR product, to determine whether cleavage has occurred or not.

**Immunoblots**

For immunoblotting, whole brain extracts or primary neuron cultures were lysed using SDS-lysis buffer (4% SDS, 20% glycerol, and 50 mM Tris-HCl, pH 6.8), and then sonicated on ice. The lysates were subsequently diluted to a concentration of 5 μg/μL. For loading purposes, an equal volume of a solution containing 0.01% bromophenol blue and 1M 2-mercaptoethanol was added to the lysates, which were then boiled at 96 °C for 3 min. An aliquot of 50 μg of denatured proteins was loaded for each experimental condition. Protein separation was performed using SDS gradient 5%–20% polyacrylamide gels (e-PAGEL, ATTO, Japan), followed by transfer onto nitrocellulose membranes (Protran, Whatman).

The nitrocellulose membrane was subjected to immunoprobing using the PCDH15 antibody (sheep polyclonal, M&D Systems, USA) and the GAPDH antibody (mouse monoclonal, MBL, Japan). Appropriate donkey secondary antibodies, namely IRDye 680RD and 800CW (LI-COR Biosciences), were utilized for detection. An infrared imager (Odyssey, LI-COR Biosciences) was employed to visualize and quantify the signals. The resulting digital data were exported in TIFF format.

**Behavioral analysis**

Open ﬁeld test was conducted as previously described [4]. Mice were placed in the center of the arena and allowed to explore the open ﬁeld (diameter: 60 cm, height: 35 cm) for the following 5 min under moderately light conditions (85 lx). The open ﬁeld was divided into an inner zone (diameter: 40 cm), and an outer zone surrounding the inner zone. The movement of mice was measured via a camera mounted above the open ﬁeld and its activity was automatically analyzed using the Ethovision automated tracking program (Brainscience Idea Co., Ltd., Osaka, Japan). Measurements included distance and time spent in the inner and outer zone.

Y-maze test was performed as previously described [5]. Each arm is 40 cm long, 12 cm high, 3 cm wide at the bottom, and 10 cm wide at the top. The arms converge in an equilateral triangular central area that is 4 cm at its longest axis. Each mouse was placed individually at the central area and allowed to move freely through the maze during an 8 min session. The series of arm entries was visually recorded. Definition of alternative behavior was successive entries into the three arms, on overlapping triplet sets. The percent alternation is calculated as the ratio of actual to possible alternations (defined as the total number of arm entries minus 2) multiplied by 100. Spontaneous alternation was associated with the capacity of short-term memory.

The elevated plus maze was conducted as described previously with minor modifications [6]. The apparatus was made of plastic material and was elevated to a height of 50 cm above the ground. Each arm of the plus maze was 16 cm in length and 10 cm in width. Additionally, the closed arms had wall enclosures that were 20 cm high. The central platform was a square of 10 × 10 cm. Light intensity around the maze was set at 100–120 lux. Mouse was placed on the elevated plus maze facing the open arm opposite to the experimenter. The number of entries and the time spent in the open and closed arms were recorded over the entire 5-min duration of the test.

Locomotor activity under a novel environment was conducted as described previously [4]. Each mouse was placed in a standard transparent rectangular rodent cage (25 × 30 × 18 cm) under moderately light conditions (15 lx). Locomotor activity was then measured for 120 min using an infrared sensor (NS-DAS-8; Neuroscience, Tokyo, Japan) placed over the cage. Locomotor activity was then measured for 120 min.

Novel object recognition test was performed as previously described with minor modifications [6] . Mice were individually habituated to an open-box (30 × 30 × 35 cm) for 3 days. During the training session, two novel objects were placed in the open ﬁeld and the animals were allowed to explore for 10 min under moderately light conditions (15 lx). The time spent exploring each object was recorded. During test sessions, one of the familiar objects used during training session was replaced by a novel object. The animals were placed back into the same box 24 h after the training session, and the mice were allowed to freely explore for 5 min. The preference index in the test session, the ratio of the amount of time spent exploring the novel object over the total time spent exploring both objects, was used to measure cognitive function. In the training session, the preference index was calculated as the ratio of time spent exploring the object that was replaced by a novel object in the test session, to the total exploration time.

The procedure for the social interaction and social novelty test were performed as previously described with minor modifications. [5] The experiment was conducted under moderately light conditions (15 lx). The social interaction chamber is a three-chambered apparatus. As doorways are built into the two dividing walls, the mice can freely access each of the three chambers. Each chamber was 20 × 40.5 × 22 cm. In habituation session, each test mouse was individually habituated to chambers. In the sociability test, small plastic container confining an unfamiliar adult male (Stranger 1) was put one of the side chambers, whereas empty small cage was placed in another side chamber. The duration of social interaction was recorded when the mouse spent within the 10 cm circle around plastic container. Subsequently the same experimental mouse was subjected to the test for social novelty, the second unfamiliar mouse being a new stranger mouse (Stranger 2) placed in the opposite side, which was previously empty during the sociability test. During social novelty test, the experimental mouse was allowed to explore all the three chambers for 10 min. The duration of social interaction with Stranger 1 or Stranger 2 was recorded in the same way as before. All stranger mice used in the experiment were WT. The entire procedures were automatically measured using the Ethovision automated tracking program (Brainscience Idea Co., Ltd., Osaka, Japan).

Prepulse inhibition (PPI) test was performed as previously described [4]. After the animals were placed in the chamber under moderately bright light conditions (180 lx) (San Diego Instruments, San Diego, CA), they were allowed to habituate for 10 min, during which 65 dB background white noise was present. The animals then received 10 startle trials, 10 no-stimulus trials, and 40 PPI trials. The intertrial interval was between 10 and 20 s and the total session lasted 17 min. The startle trial consisted of a single 120 dB white noise burst lasting 40 ms. PPI trials consisted of a prepulse (20 ms burst of white noise at 69, 73, 77, or 81 dB intensity) followed, 100 ms later, by the startle stimulus (120 dB, 40 ms white noise). Each of the four prepulse trials (69, 73, 77 or 81 dB) was presented 10 times. Sixty different trials were pseudorandomly presented, ensuring that each trial was presented 10 times and that no two consecutive trials were identical. The resulting movement of the animal in the startle chamber was measured for 100 ms after startle stimulus onset (sampling frequency 1 kHz), rectiﬁed, ampliﬁed and fed into a computer, which calculated the maximal response over the 100-ms period. Basal startle amplitude was determined as the mean amplitude of the 10 startle trials. PPI was calculated according to the formula: 100 × [1 − (PPx/P120)]%, in which PPx was the mean of the 10 PPI trials (PP69, PP73, PP75, or PP80) and P120 was the basal startle amplitude.

Fear conditioning test was performed as previously described [7]. In the conditioning phase, each mouse is placed in the training cage (30 × 30 × 40 cm) equipped with a metal floor, and a 15-s tone (85 dB) is delivered (conditioned stimulus). During the last 5 s of the tone stimulus, a foot shock of 0.8 mA is delivered as an unconditioned stimulus through a shock generator. This procedure is repeated four times with 15-s intervals. Following the 24 h conditioning, context-dependent test was carried out. For context-dependent test, mouse is placed in the training cage, and the freezing response is measured for 2 min in the absence of the conditioned stimulus. Following the 4 h context-dependent test, tone-dependent test was conducted. For tone-dependent test, the freezing response was measured in the neutral cage for 1 min in the presence of a continuous-tone stimulus identical to the conditioned stimulus using mice which had been subjected to context-dependent test.

Light/dark (LD) transition test was conducted as previously described with minor modifications [8]. The apparatus used for the LD transition test consisted of a cage (15 ×  15  ×  15 cm) divided into two sections of equal size by a partition containing a door. One chamber was brightly illuminated (300 lux) whereas the other chamber was darker than 10 lux. Mice were placed into the dark side and allowed to move freely between the two chambers with the door open for 10 min. The time spent in each side and activity were recorded by using a MED-PC IV (Med Associations, Inc., St Albans City, USA).

Rotarod test was performed according to a previous report with minor modifications [9]. In brief, Rotarod test was performed using the MK-600 (MUROMACHI KIKAI CO., LTD, Tokyo, Japan), under moderately light conditions (15 lux). The mice were trained for 3 days. During the training session, the mice were placed on a rod rotating at 6 revolutions per minute (rpm) and the time taken for them to fall from the rod was measured. If a mouse stayed on the rod until the end of the 2-min trial, a time of 120 s was recorded. Test session was performed on day 4. The mice were placed on a rod rotating at 12 rpm, and the time taken for them to fall from the rod was measured. Each mouse was subjected to 6 trials per day with a 15 min intertrial interval in the training and test session. We recorded the score the average value in a set of measurements. The apparatus was routinely cleaned with water and ethanol following each session.

**24-h locomotor activity test**

Male *Pcdh15-*null, *Pcdh15-*het, and WT mice were bred via *in vitro* fertilization from *Pcdh15-*het parents. At 8 weeks of age, the mice were acclimated to the apparatus used for the locomotor activity test but with the measurement period extended to 24 h. The locomotor activity of the mice was measured every 5 min during the 24 h period using digital counters equipped with infrared sensors (BrainScience Idea, Osaka, Japan). For the initial nine hours (hours 0–9), the lights were on and were off for the subsequent 12 hours (hours 16–21). The lights were on again for the last three hours (hours 22–24). The mice were habituated to the test environment for 120 min (the habituation period) before beginning the measurement of locomotor activity.

**Hematoxylin and eosin (HE) staining**

Mice were deeply anesthetized with 2,2,2-Tribromoethanol (Avertin) and intracardially perfused with phosphate-buffered saline (PBS) and 4% paraformaldehyde in PBS. The fixed brains were paraffin-embedded, 5μm-thick sections were prepared, immersed in hematoxylin–eosin solution (Muto Pure Chemicals Co. Ltd, Japan), and stained.

**Immunohistochemistry**

The mice were anesthetized with 2,2,2-Tribromoethanol and intracardially perfused on ZT7 or ZT13 with 4% paraformaldehyde/PBS, and the whole brain was removed. The brain sections (50 μm) prepared with vivratome were incubated with 20% normal donkey serum in PBS containing 0.2% Triton-X 100 (Tx) (blocking buffer) for 1 h at room temperature (RT) and then treated with rabbit polyclonal antibody against c-Fos (1:500; CAT#2250, Cell Signaling Technology., MA, USA) diluted in blocking buffer for 1 day at 4°C. The sections were then incubated with the Alexa 488-conjugated secondary antibody (1:2000; Thermo Fisher Scientific Inc., Suwannee, GA) diluted in Can Get Signal immunostain solution A (TOYOBO CO., LTD., Osaka, Japan) for 2–3 h at RT. The morphology of each section was analyzed using a laser scanning confocal microscope (TiE-A1R, Nicon Solutions Co., Ltd., Tokyo, Japan).

**Phase analysis**

Locomotor activity phase analysis was performed using activity data collected under LD cycles, following the methods based on previous research [10]. Activity onsets and offsets were determined by the first upward and the last downward threshold crossings of the moving averaged (3-h window) locomotor activity data during ZT9-ZT27 for each day, respectively. The threshold was set as the time series of the moving averaged (24-h window) locomotor activity data. The candidates of activity midpoints were defined as the points at which total locomotor activity in the preceding 8 h was the same as total locomotor activity in the subsequent 8 h. Then, the first candidate during ZT9-ZT27 was defined as the activity midpoint. Activity peak was defined as the time point of the maximum value of the moving averaged (1-h window) locomotor activity during ZT9-ZT27. These calculations were conducted with custom-made Python codes.

**Mapping of mouse brain activity through an automated volume analysis of c-Fos**

This protocol was provided by Certerra Inc., US.

**(i) Light-sheet fluorescence microscopy imaging**

The brains were immunostained and chemically cleared following our published iDISCO + protocol [11], and subsequently imaged in the sagittal orientation (right lateral side up) on a light-sheet fluorescence microscope (Ultramicroscope II, LaVision Biotec) equipped with an sCMOS camera (Andor Neo) and a 4×/0.5 objective lens (MVPLAPO 4×) with a 6-mm-working-distance dipping cap. Version v144 of the Inspector Microscope controller software was used. The samples were scanned with a 5-μm step size using the continuous light-sheet scanning method with the included contrast blending algorithm for the 640 and 595 nm channels (20 acquisitions per plane), and without horizontal scanning for the 480-nm channel.

**(ii) Statistical methods used for the identification of differences between groups**

Statistical comparisons between the different groups were run based on either regions of interest (ROIs) or evenly spaced voxels. Voxels are overlapping 3D spheres with a diameter of 100 μm each and spaced 20 μm apart from each other. The cell counts at a given location, Y, were assumed to follow a negative binomial distribution, with the mean being linearly correlated with one or more experimental conditions, X: E[Y] = α + βX. For example, when testing an experimental group vs. a control group, the X was a single column showing the categorical classification of the mouse sample to group id, i.e., 0 for the control group and 1 for the experimental group. We found the maximum likelihood coefficients α and β through iterative reweighted least squares, obtaining estimates for sample standard deviations in the process, from which we obtained the significance of the β coefficient. A significant β means that the group status is related to the cell count intensity at the specified location. The z-values in our summary tables corresponded to this β coefficient normalized by its sample standard deviation, which, under the null hypothesis of no group effect, had an asymptotic standard normal distribution. The p-values provide us the probability of obtaining a β coefficient as extreme as the one observed by chance, assuming that this null hypothesis was true. In the case of three (or more) groups, we utilized the Tukey’s honest significance test to adjust the p-values of the group factor coefficients, to control for multiple comparisons: group1v2, group1v3, and group2v3. To account for multiple comparisons across all voxel/ROI locations, we performed thresholding of the p-values and reported the false-discovery rates (FDR) using the Benjamini–Hochberg procedure. Rather than correcting for type I error rates, this method controls the number of false positives among the tests that have been deemed significant.

**(iii) Statistical methods used for the identification of similarity to Certerra’s Clinical Pharmacomap Library**

The comparative clustergram was based on pharmacomaps generated as voxelized statistical significance maps using negative binomial regression, which compared the c-Fos + cell densities between the drug and control groups. Two subsequent FDR corrections of 0.05 and 0.001 were used for each drug. These FDR (q) values were used to threshold the statistical significance maps and create a 3-value significance map of voxel excitation (+1), no activation (0), and voxel inhibition (–1). The distance measurement used for similarity between drugs was based on an extended version of dice similarity metric that accounts for the trinary activation profile of the drug. The similarity metric was as follows:

*Modified Dice Similarity* = ((2 × ((A+ ∩ B+)∪ (A– ∩ B–))))/((|A+|∪|B+|∪|A–|∪|B–|)),

with the variables as follows:

A+ and B+ are a set of all excited voxels above the q+ value of the respective drug; and A– and B– are a set of all inhibited voxels below the q-value of the respective drug. From the similarity metric, the distance was calculated as follows:

*Similarity Distance* = 1 − *Modified Dice Similarity*

Using this metric, a similarity distance matrix for each drug against every other drug was calculated, and the distance matrix was constructed as follows: the x and y axis were used as the list of different drugs with their q-value threshold for their dosages. This distance map was then used for hierarchical clustering. In all cases, as expected, the different q-values of the same drug and same dosage clustered at the first level, typically followed by the clustering of the same drug at different dosages, and then clustering of drugs based on the similarity of the pharmacomaps.

**Preparation of total RNA**

Eleven-week-old *Pcdh15-*null and WT mice were euthanized via cervical dislocation, and their brains were swiftly extracted. The visual cortical areas were subsequently dissected and rapidly frozen using liquid nitrogen for preservation. Upon thawing, lysates were generated with Trizol (Thermofisher, US), and total RNA was dissolved in RNase-free sterile water. The quantification of RNA was performed, and 1 μg of RNA was allocated for RT-PCR.

**RT-PCR**

A total of 1 μg of total RNA was utilized for the Superscript III RT cDNA synthesis kit (Thermofisher, US) to carry out reverse transcription. Subsequent RT-PCR was conducted. The primer sequences employed for gene detection were as follows: mFos (c-fos): 5'- gggaatggtgaagaccgtgtcaggag -3', 5'- gcagccatcttattccgttcccttcg -3'; beta-actin (control): 5'-gactgggatcatttgttcacacatgcag-3', 5'-ggcccttgaacttcggtatctacactgag-3'.

**Quantitative RT-PCR (qPCR)**

Quantitative expression analysis using qPCR was performed on a QuantStudio5 instrument (Applied Biosystems) with the KAPA SYBR Fast qPCR kit (KAPA Biosystems, US) [12]. The primer sequences used for gene detection were as follows: mFos (c-fos): 5'- gggaatggtgaagaccgtgtcaggag -3', 5'- gcagccatcttattccgttcccttcg -3'; m18S (control): 5'- gcaattattccccatgaacg -3', 5'- ggcctcactaaaccatccaa -3'.

**II. Supplementary Figures**

**Figure S1. Generation of *Pcdh15*-deletion mice and expression profiles of PCDH15 protein**

(a) T7E1 assay for the optimization of guide RNA in CRISPR/Cas9 cleavage. (b) Results of the number of eggs/embryos in the process of generating *Pcdh15-*deletion mice. Injections were performed on the nuclei of all 112 fertilized eggs, resulting in the successful obtainment of two littermates with the designed knock-in sequence. (c) Genotyping results of *Pcdh15*-deletion mice through PCR and EcoRI restriction enzyme digestion. PCR products from knockout alleles can be cleaved with EcoRI. (d) Immunoblot analysis utilizing 50 μg of total lysates from various organs of WT adult mice for the detection of PCDH15 protein. GAPDH was employed as an internal control. (e) Immunoblot analysis using 100 μg of total lysates prepared from various developmental stages of WT to detect PCDH15 protein. (f) Immunoblot analysis using 10 μg of total lysates from *in vitro* cortical cultured neurons for the detection of PCDH15 protein. (g) Hematoxylin and eosin (HE) staining of coronal sections from the adult brain of *Pcdh15*-null and WT.

**Figure S2. General behavioral analysis of *Pcdh15-*het**

This supplementary figure complements Figure 2 and Table 1. It depicts the performance of *Pcdh15-*het mice in various behavioral tests: open field test (a), Y-maze test (b), elevated plus maze test (c), rotarod test (d), novel object recognition test (e), social interaction test (f), and fear conditioning test (g). Data are expressed as mean ± standard error of the mean (SEM) (WT males, n = 15; *Pcdh15-*het males, n = 15).

**Figure S3.** **24-h locomotion activity test**

(a) We continuously conducted locomotor activity tests for 24 h on WT male (N = 12), *Pcdh*15-het male (N = 12), and *Pcdh15-*null male (N = 5) mice individually and plotted the average activity level per hour. (b) The period from 10 AM to noon served as the habituation time. The illuminated period, from noon to 9 PM (Daytime 1), the dark period from 9 PM to 9 AM of the next day (Nighttime), and the illuminated period from 9 AM to noon (Daytime 2) were divided into three segments. The data are presented as mean ± standard error of the mean (SEM). Statistical comparisons were made using two-way analyses of variance (ANOVA) with Bonferroni post-hoc tests [13]. All statistical tests were performed using GraphPad Prism v. 9.0 (GraphPad Software Inc., San Diego, CA, USA). Nighttime (lights-off) activity in *Pcdh15-*null was significantly greater than that in the WT or *Pcdh15*-het mice.

**Figure S4. Continuous activity and body temperature data in mice with abdominally implanted nano tag**

Individual mouse data contributing to Figure 3 are displayed. Plotted using nano tag Viewer software. The horizontal axis represents the 24-h time frame, while the vertical axis presents the activity (histogram) and body temperature (line graph) for each day. Three-dimensional surface plots depicting changes in activity and temperature over the entire duration were generated using MATLAB software (Mathworks, Japan).

**Figure S5. Phase analyses of locomotor activity using nano tag**

(a) Phase analysis. Average onset, offset, midpoint, and peak time (depicted in ZT) of locomotor activity phase for WT (white bar, n = 4) and *Pcdh15*-null (black bar, n = 4) were calculated as described in the methods. (b) Activity counts. The average of activity counts per 5 min for WT (white bar, n = 4) and *Pcdh15*-null (black bar, n = 4) were calculated for the entire 56-day recordings. (c) Ratio of inactive time. The average ratio of inactive periods defined as 5 min interval with less than 5 counts of activity of WT (white bar, n = 4) and *Pcdh*15-null (black bar, n = 4) was calculated over the entire 56-day recordings.

Bars indicate SEM.

**Figure S6. Various studies on c-Fos expression in the brain**

(a) Three-dimensional constructed images of stacked q-value voxel maps comparing WT and *Pcdh15-*null. Left corresponds to ZT13, and right to ZT7. Generated using ImageJ (NIH, US). (b) c-Fos positive cell counts throughout the entire brain as a result of automated analysis (N = 3 for each group). Data are presented as mean ± SEM. (∗∗p < 0.01). Two-way repeated measures ANOVA analyzed the effect of Pcdh15-deletion, sampling time, and their interaction, with sampling time showing significance at p <0.001 (Gene deletion: F(1, 8) = 0.64; Time: F(1,8) = 50.33; Time × Gene deletion: F(1,8) = 1.48). (c) Images of c-Fos immunostaining around the VISp area. Captured within the red frame of the lower right atlas image. Scale bars represent 200 μm. (d, e) Results of quantitative RT-PCR for *c-fos* expression (e) Relative expression of *c-fos*. Data represent mean ± SEM (N = 5 for each group). Two-way repeated measures ANOVA analyzed the effect of Pcdh15-deletion, sampling time, and their interaction, all of which were significant at p <0.0001 (Gene deletion: F(1, 8) = 56.6; Time: F(1,8) = 233.5; Time × Gene deletion: F(1,8) = 63.2). (a) 3D constructed image of stacked q-value voxel maps comparing WT and *Pcdh15*-null. Left is ZT13 and right is ZT7. These are generated by ImageJ (NIH, US). (b) c-Fos positive cell counts in whole brain in result of automated analysis. (N = 3 for each group). Data represent the mean ± SEM. (∗∗p < 0.01). Two-way repeated measures ANOVA examined the *Pcdh15*-deletion, sampling time, and the interaction between the two, sampling time was significant at p <0.001 (Gene deletion: F(1, 8) = 0.64; Time: F(1,8) = 50.33; Time × Gene deletion: F(1,8) = 1.48). (c) Images of c-Fos immunostaining around VISp. The images were captured around the red frame of the lower right atlas image. Scale bars are 200μm. (d, e) Result of quantitative RT-PCR of *c-fos* expression. (e) Relative expression of *c-fos.* Data represent the mean ± SEM (N = 5 for each group). Two-way repeated measures ANOVA examined the *Pcdh15*-deletion, sampling time, and the interaction between the two, all of which were significant at p <0.0001 (Gene deletion: F(1, 8) = 56.6; Time: F(1,8) = 233.5; Time × Gene deletion: F(1,8) = 63.2).

**Supplementary Table 1. Comparison of c-Fos-positive cell counts between WT and Pcdh15-null at ZT13**

| ROIs | WT | | *Pcdh15*-null | | z | p-value | q-value |
| --- | --- | --- | --- | --- | --- | --- | --- |
|  | mean | S.D. | mean | S.D. |  |  |  |
| MOs | 408.7 | 189.9 | 837.3 | 113.3 | 3.13 | 0.001731 | 0.027666 |
| SSp-ll2/3 | 109.0 | 20.1 | 213.3 | 27.0 | 6.67 | 2.64E-11 | 7.93E-09 |
| AUDp6b | 179.0 | 22.5 | 83.0 | 23.4 | −5.74 | 9.34E-09 | 0.000002 |
| VISp | 2007.0 | 1055.6 | 5249.0 | 1752.4 | 3.13 | 0.001726 | 0.027666 |
| PL | 87.7 | 26.5 | 178.3 | 52.8 | 3.52 | 0.000428 | 0.009879 |
| ILA | 152.7 | 43.9 | 260.7 | 44.1 | 3.29 | 0.000997 | 0.018258 |
| ORB | 216.7 | 40.8 | 421.3 | 91.7 | 4.85 | 0.000001 | 0.000086 |
| LA | 574.3 | 54.4 | 360.7 | 88.1 | −3.65 | 0.000259 | 0.007666 |
| BLAv | 165.0 | 19.1 | 119.7 | 20.6 | −3.33 | 0.000856 | 0.016556 |
| CEA | 915.7 | 15.3 | 584.0 | 56.0 | −10.90 | 1.20E-27 | 1.44E-24 |
| GPe | 655.3 | 344.3 | 191.0 | 77.9 | −3.43 | 0.000613 | 0.013224 |
| BST | 278.3 | 32.7 | 503.0 | 180.4 | 3.37 | 0.000763 | 0.015003 |
| PVZ | 270.0 | 11.5 | 381.7 | 74.4 | 3.58 | 0.000347 | 0.008864 |
| VMH | 148.3 | 52.0 | 68.7 | 18.9 | −3.73 | 0.000193 | 0.005919 |
| LZ | 7695.3 | 1218.5 | 5650.3 | 643.3 | −3.33 | 0.000883 | 0.016797 |
| SNr | 1745.0 | 103.9 | 1157.0 | 267.8 | −3.56 | 0.000366 | 0.009135 |
| NLL | 768.0 | 254.9 | 329.7 | 37.6 | −4.97 | 0.000001 | 0.000051 |
| DTN | 77.3 | 17.0 | 119.7 | 13.3 | 4.05 | 0.000051 | 0.002038 |
| NI | 101.3 | 13.5 | 169.7 | 39.3 | 3.97 | 0.000071 | 0.002734 |
| CN | 656.0 | 371.3 | 218.0 | 54.6 | −3.39 | 0.000700 | 0.014226 |

**Supplementary Table 2. Comparison of c-Fos-positive cell counts between WT and Pcdh15-null at ZT7**

| ROIs | WT | | *Pcdh15*-null | | z | p-value | q-value |
| --- | --- | --- | --- | --- | --- | --- | --- |
|  | mean | S.D. | mean | S.D. |  |  |  |
| CTX | 46793.7 | 32826.4 | 12320.0 | 4534.7 | −3.64 | 0.000271 | 0.002874 |
| AUD | 2775.3 | 2468.5 | 669.0 | 147.8 | −3.32 | 0.000886 | 0.007176 |
| VIS | 13674.0 | 13813.5 | 1214.7 | 554.2 | −4.64 | 0.000003 | 0.000085 |
| RSPagl | 420.3 | 287.0 | 47.0 | 8.9 | −5.32 | 1.05E-07 | 0.000004 |
| TEa | 3777.7 | 3072.8 | 569.7 | 241.7 | −4.17 | 0.000030 | 0.000527 |
| ECT | 4542.0 | 2632.4 | 703.3 | 262.6 | −5.79 | 0.000000 | 0.000000 |
| PIR | 1158.3 | 687.6 | 330.7 | 191.1 | −3.31 | 0.000934 | 0.007467 |
| PAA | 123.7 | 91.5 | 24.7 | 23.8 | −2.99 | 0.002745 | 0.017775 |
| CA3 | 867.3 | 347.3 | 402.3 | 189.5 | −2.59 | 0.009530 | 0.046832 |
| DG | 1989.3 | 355.7 | 927.0 | 274.8 | −4.74 | 0.000002 | 0.000058 |
| ENT | 3843.7 | 1955.5 | 1379.3 | 626.0 | −3.11 | 0.001862 | 0.013288 |
| LA | 293.0 | 144.9 | 55.7 | 18.8 | −5.17 | 2.34E-07 | 0.000009 |
| BLA | 387.0 | 148.3 | 90.3 | 64.1 | −4.08 | 0.000044 | 0.000697 |
| BMA | 391.3 | 231.8 | 64.7 | 18.0 | −5.96 | 2.47E-09 | 1.85E-07 |
| CEA | 283.3 | 86.8 | 128.3 | 42.1 | −3.83 | 0.000128 | 0.001595 |
| MEA | 367.7 | 69.9 | 104.3 | 47.6 | −5.20 | 1.96E-07 | 0.000008 |
| LGv | 179.7 | 79.8 | 20.7 | 9.5 | −6.77 | 1.25E-11 | 3.76E-09 |
| HY | 1858.3 | 1253.5 | 717.7 | 231.0 | −2.68 | 0.007357 | 0.038860 |
| SCm | 1491.0 | 936.4 | 388.7 | 219.1 | −3.45 | 0.000553 | 0.004910 |
| CN | 298.3 | 137.7 | 67.3 | 14.6 | −5.80 | 6.81E-09 | 4.16E-07 |

**Movie S1. Marked hyperlocomotion in *Pcdh15*-null**

Video showing free-moving WT, *Pcdh15*-het, and *Pcdh15*-null 12-week-old male mice.

**III. Supplementary References**

1. Sekiguchi, M, Sobue A, Kushima I, Wang C, Arioka Y, Kato H *et al.* ARHGAP10, which encodes Rho GTPase-activating protein 10, is a novel gene for schizophrenia risk. *Transl Psychiatry* 2020; 10: 247.

2. Sawahata, M, Mori D, Arioka Y, Kubo H, Kushima I, Kitagawa K *et al.* Generation and analysis of novel Reln-deleted mouse model corresponding to exonic Reln deletion in schizophrenia. *Psychiatry Clin Neurosci* 2020; 74: 318-327.

3. Aida, T, Chiyo K, Usami T, Ishikubo H, Imahashi R, Wada Y *et al.* Cloning-free CRISPR/Cas system facilitates functional cassette knock-in in mice. *Genome Biol* 2015; 16: 87.

4. Ibi, D, Nagai T, Kitahara Y, Mizoguchi H, Koike H, Shiraki A *et al.* Neonatal polyI:C treatment in mice results in schizophrenia-like behavioral and neurochemical abnormalities in adulthood. *Neurosci Res* 2009; 64: 297-305.

5. Alkam, T, Kim HC, Hiramatsu M, Mamiya T, Aoyama Y, Nitta A *et al.* Evaluation of emotional behaviors in young offspring of C57BL/6J mice after gestational and/or perinatal exposure to nicotine in six different time-windows. *Behav Brain Res* 2013; 239: 80-89.

6. Nagai, T, Takuma K, Kamei H, Ito Y, Nakamichi N, Ibi D *et al.* Dopamine D1 receptors regulate protein synthesis-dependent long-term recognition memory via extracellular signal-regulated kinase 1/2 in the prefrontal cortex. *Learn Mem* 2007; 14: 117-125.

7. Ibi, D, Nagai T, Koike H, Kitahara Y, Mizoguchi H, Niwa M *et al.* Combined effect of neonatal immune activation and mutant DISC1 on phenotypic changes in adulthood. *Behav Brain Res* 2010; 206: 32-37.

8. Matsuo, N, Takao K, Nakanishi K, Yamasaki N, Tanda K & Miyakawa T. Behavioral profiles of three C57BL/6 substrains. *Front Behav Neurosci* 2010; 4: 29.

9. Okun, E, Barak B, Saada-Madar R, Rothman SM, Griffioen KJ, Roberts N *et al.* Evidence for a developmental role for TLR4 in learning and memory. *PLoS One* 2012; 7: e47522.

10. Tam, SKE, Brown LA, Wilson TS, Tir S, Fisk AS, Pothecary CA *et al.* Dim light in the evening causes coordinated realignment of circadian rhythms, sleep, and short-term memory. *Proc Natl Acad Sci U S A* 2021; 118.

11. Renier, N, Adams EL, Kirst C, Wu Z, Azevedo R, Kohl J *et al.* Mapping of Brain Activity by Automated Volume Analysis of Immediate Early Genes. *Cell* 2016; 165: 1789-1802.

12. Arioka, Y, Shishido E, Kushima I, Suzuki T, Saito R, Aiba A *et al.* Chromosome 22q11.2 deletion causes PERK-dependent vulnerability in dopaminergic neurons. *EBioMedicine* 2021; 63: 103138.

13. Elder, GA, Ragnauth A, Dorr N, Franciosi S, Schmeidler J, Haroutunian V & Buxbaum JD. Increased locomotor activity in mice lacking the low-density lipoprotein receptor. *Behav Brain Res* 2008; 191: 256-265.
